# The Biodemography of Fertility: A Review and Future Research Frontiers

**DOI:** 10.1007/s11577-015-0319-4

**Published:** 2015-09-21

**Authors:** Melinda C. Mills, Felix C. Tropf

**Affiliations:** 1 Department of Sociology and Nuffield College, University of Oxford, 1 New Road, OX1 1NF Oxford, UK; 2 Department of Sociology, Interuniversity Center for Social Science Theory and Methodology (ICS), University of Groningen, Grote Rozenstraat 31, 9712 Groningen, TG The Netherlands

**Keywords:** Fertility, Age at first birth, Number of children ever born, Genetics, Behavioural genetics, Molecular genetics, Natural selection, Fertilität, Alter bei Erstgeburt, Endgültige Kinderzahl, Genetik, Verhaltensgenetik, Molekulargenetik, Natürliche Auslese

## Abstract

The social sciences have been reticent to integrate a biodemographic approach to the study of fertility choice and behaviour, resulting in theories and findings that are largely socially-deterministic. The aim of this paper is to first reflect on reasons for this lack of integration, provide a review of previous examinations, take stock of what we have learned until now and propose future research frontiers. We review the early foundations of proximate determinants followed by behavioural genetic (family and twin) studies that isolated the extent of genetic influence on fertility traits. We then discuss research that considers gene and environment interaction and the importance of cohort and country-specific estimates, followed by multivariate models that explore motivational precursors to fertility and education. The next section on molecular genetics reviews fertility-related candidate gene studies and their shortcomings and on-going work on genome wide association studies. Work in evolutionary anthropology and biology is then briefly examined, focusing on evidence for natural selection. Biological and genetic factors are relevant in explaining and predicting fertility traits, with socio-environmental factors and their interaction still key in understanding outcomes. Studying the interplay between genes and the environment, new data sources and integration of new methods will be central to understanding and predicting future fertility trends.

## Abstract (de)

In sozialwissenschaftlichen Untersuchungen von Fertilitätsentscheidungen und –verhalten sind biodemographische Ansätze bislang kaum integriert worden, was zu überwiegend sozial-deterministischen Theorien und Befunden geführt hat. Der vorliegende Beitrag diskutiert zunächst die Gründe für diese weitgehend fehlende Integration, gibt dann einen Überblick über frühere Untersuchungen, fasst die bisher vorliegenden Erkenntnisse zusammen und zeigt schließlich Perspektiven zukünftiger Forschung auf. Ausgehend von grundlegenden Arbeiten zu den proximalen Determinanten der Fertilität werden verhaltensgenetische (Familien- und Zwillings-)Studien besprochen, die das Ausmaß genetischer Einflüsse auf Fertilitätsmerkmale isolieren konnten. Anschließend wird die Forschung zur Gen-Umwelt-Interaktion, zur Bedeutung von kohorten- und länderspezifischen Analysen sowie von Modellen, die Bildung und Fertilitätsentscheidungen vorausgehende Motivlagen berücksichtigen, betrachtet. Darüber hinaus werden Möglichkeiten und Grenzen molekulargenetischer Studien besprochen, bevor schließlich ein kurzer Überblick über Arbeiten aus der evolutionären Anthropologie und Biologie mit einem Fokus auf den Aspekt der natürlichen Auslese gegeben wird. Es zeigt sich, dass biologische und genetische Faktoren für das Verständnis und die Vorhersage von Fertilitätsmerkmalen relevant sind und dass ihre Interaktion mit sozialen Umweltfaktoren zentral für das Verständnis von Fertilitätsoutcomes ist. Für die Vorhersage zukünftiger Fertilitätstrends wird die Untersuchung des Zusammenspiels von Gen-Umwelt-Faktoren sowie die Nutzbarmachung neuer Datenquellen und die Integration neuer Methoden eine wesentliche Rolle spielen.

## Introduction

Fertility research within demography and the social sciences has been largely dominated by social science or environmental explanations of fertility behaviour and outcomes (Balbo et al. 2013). Yet a growing body of research over the last decades has demonstrated the relevance of including biological and genetic factors into our understanding of fertility outcomes (e.g., Udry 1996; Kohler et al. 1999, 2002, 2006; Foster 2000; Wachter and Bulatao 2003; Kohler and Rodgers 2003; Haaga 2003; Miller et al. 2010). Recent advances in biology, molecular genetics, medical sciences, reproductive medicine and evolutionary anthropology have likewise increased the relevance of adopting a biodemographic approach to study fertility in sociology and demography. Biodemography is a recent branch of science that integrates biology and demography, focussing on the complementary biological and demographic determinants of and interactions between the birth and death processes as they relate to populations, and often to humans in particular (Carey and Vaupel 2005).

The aim of this paper is to first reflect on past challenges that slowed the integration of a biodemographic approach to fertility research, provide a review of previous examinations, take stock of what we have learned until now and propose future frontiers for promising research. Our work draws primarily on research carried out within demography and sociology, but with attention to more recent work particularly in the areas of evolutionary anthropology, and behavioural and molecular genetics. Given the fact that this is a general review article aimed at a social science audience, we are often very explicit in our explanation of central concepts in this area of research (e.g., heritability, SNPs) and take an effort to describe methods that are less familiar to social scientists (e.g., twin models, GWAS) in the text and accompanying Appendices.

We have opted to organize our scientific history of the biodemography of fertility around several themes. We first review the main reasons for the lack of attention—and in some cases outright resistance—to adopting a biodemographic approach to fertility research. We then discuss the early foundations of combining a biological and behavioural approach to the study of fertility via the use of proximate determinants. Next, we turn to a review of research that has adopted a behavioural genetic approach to determine whether fertility has a genetic component, often using family and twin study designs. This is followed by more recent research in the area of molecular genetics, which shifts from identifying whether there is a genetic component to fertility to isolating where it is located. Anthropological and evolutionary approaches are then touched upon. The paper concludes with a broader discussion that reflects upon what we have learned until now and potential future fertile frontiers in this area of research. Finally, Appendices and Footnotes provide more detailed explanations of the central terms used in this paper which may be unfamiliar to a social science audience.

## Defining fertility

Before embarking upon this review, it is useful to first define the terminology related to ‘fertility’ as it often differs across disciplines. Throughout this paper we generally refer to human fertility and not cross-species research. It is also essential to note that the broader terms fertility and infertility have different meanings in demography, evolutionary biology and reproductive medicine (Mills et al. 2011). In demography and related social sciences, ‘fertility’ refers to performance and the bearing and timing of live births. The focus is often on the two interrelated terms of the *tempo* (or timing) of childbearing and the *quantum* or actual number of children that women have during a certain period. Throughout this study, we also often refer to two of the most central indicators studied in this field until now, which is the tempo measure of age at first birth (AFB) and the quantum measure of number of children ever born (NEB).

In demography and sociology, quantum is often referred to as the number of children (e.g., Kohler et al. 1999) whereas in biological research the same outcome is referred to life-time reproductive success (Byars et al. 2010) or the number of offspring (Zietsch et al. 2014). In evolutionary research, fertility quantum defines the key term of ‘fitness’,^1^ which is a function of the number of children of a subject in relation to the number of children of peers of the same birth cohort (e.g., Kirk et al. 2001; Stearns et al. 2010). This in turn is used to measure how far the fertility quantum leads to relatively higher chances to successfully transmit genes to the next generation. Due to improvements in hygiene and the reduction in prenatal, infant and child mortality in industrialized societies, NEB has emerged as the gold standard to measure lifetime reproductive success indicating biological fitness (Stearns et al. 2010). Another distinction that differs between disciplines is the terminology related to infertility and fecundity. In reproductive medicine, infertility denotes the (in)ability of couples, women or men, to conceive and have children given unprotected intercourse, whereas in demography and sociology, this refers to infecundity or sterility.

## Why is a biodemographic approach to fertility less prevalent?

Although there has been some recognition of the biology underlying fertility, sociologists and demographers have been reticent, in adopting and integrating findings and approaches from behavioural and molecular genetics, neuroendrocrinology and cross-species life history analysis (Wachter 2003). Although not exhaustive, we outline some central reasons for this avoidance.

A first historical reason, also noted by others (Kohler et al. 1999; Rodgers et al. 2001), is that due to Fisher’s (1930) Fundamental Theorem of Natural Selection, genetically informed fertility research has been neglected. Fisher’s theory states that fertility is a fitness trait, which theoretically entails that heritability should be zero. As we demonstrate shortly, however, a series of studies produced evidence that this is not the case.

A second prominent reason for the avoidance of research that adopts a behavioural genetic approach in the social sciences in general—but particularly in relation to fertility—is the dark history related to eugenic policies that emerged in the 1880s and subsequent extreme atrocities in recent history. Eugenics focused on so-called ‘improving’ humanity via supposedly scientific methods that proposed selective breeding. As Levine and Bashford (2010, p. 3) describe, the aim of the eugenics movement was “to affect reproductive practice through the application of theories of heredity.” The aim was to prevent life (sterilization, contraception, abortion), ‘fitter’ life (training, rearing of children, public health) and promote pronatalist goals, but also at its most extreme, to end life (so-called euthanasia of the disabled) (Levine and Bashford 2010). As a result of these policies, hundreds of thousands of people were segregated and sterilized or lost their lives. This perspective has been widely, and rightly, condemned. It is essential to note that the type of research described in this paper and within the mainstream of contemporary peer-reviewed research in behavioural and molecular genetics has no eugenic goals or ties. Considering this grave history and link of eugenics and fertility, it remains important to explicitly acknowledge this point with the goal to prevent similar abuses to ever occur in the future.

Third, as noted in a *Population Studies* article by Thoday et al. in 1970, social scientists often ignore biology and genetics (and vice versa) due to a lack of understanding and training in their concepts and methods and virtually no communication and cooperation between disciplines, which arguably still holds today almost 50 years later. As touched upon shortly, the growth of candidate gene studies in the social sciences came at around the same time that they were shown to be an incorrect method that produced false positives in genetics (Ioannidis 2005). The lack of interdisciplinary research teams and funding has resulted in parallel literatures and disciplines that operate virtually in a complete vacuum of one another.

Fourth, although this has rapidly started to change, the survey and registration data mainly used by social scientists to study fertility has generally lacked any biomarker or genetic measures. Conversely, if combined genetic and register or survey data is available, many of the medical or genetic cohort datasets only include very crude measures of core social science and environmental indicators even though they are likely pivotal in understanding gene and socio-environment interactions (e.g., education, occupational or marital history, social capital and networks).

This reluctance, lack of data and interdisciplinary training that combines strong social and biological or genetic measures has resulted in fertility theories and explanations in the social sciences that are generally socially deterministic, often solely based on explanations related to agency, motivation, conscious choice and intentions (for a review see: Balbo et al. 2013), which are in turn highly conditioned by the environments of the family, peers, organizations, local and national institutional contexts (Mills and Blossfeld 2005). Increasing evidence demonstrates that choice, agency and the behavioural outcomes that we examine in fertility, however, are not only socially-determined, but also linked to an individual’s genetic architecture and beyond, such as proteins, hormones, neurons, gametes and other factors (Udry 1996; Wachter 2003, 2008; Freese 2008). Furthermore, recent research demonstrates that as Udry (1996) and others hypothesized some time ago, that a portion of the genetic influence of fertility is related to the motivational precursors to fertility (Miller et al. 2010). In other words, integrating genetics into the analysis of fertility will significantly improve our understanding and the explanatory power of our models. It is important to stress that biological explanations and heritability should not be mistaken for genetic determinism. As we describe shortly, the link between genes and phenotypic traits or outcomes may be indirect and mediated through environmental and psychological factors. This review will argue towards an integrative approach across disciplines.

## The foundations: proximate determinants

A biological approach within contemporary demography was initially introduced to the study of fertility by Davis and Blake (1956) with the proximate determinants of fertility, which was later refined by Bongaarts (1978, 1982) and Bongaarts and Potter (1983). These seven factors that include both biological and behavioural determinants have been highly influential in shaping our theory and understanding of human fertility in demography. These are: (1) proportion of married women among all women of reproductive age (as a proxy to capture women exposed to sexual intercourse, also more broadly measured later by percentage of women in a sexual union, frequency of sexual intercourse), (2) contraceptive use effectiveness, (3) duration of postpartum infecundability (or postpartum insusceptibility),^2^ (4) induced abortion, (5) fecundability, (6) prevalence of permanent sterility; and, (7) spontaneous intrauterine mortality. The first four were considered as the most prominent proximate determinants since they differ greatly between populations and due to the fact that fertility is the most sensitive to changes in these measures.

Criticisms and revisions of the model acknowledged the need to include sexual activities outside of marriage and higher levels of primary and secondary sterility in certain regions (see for e.g., Stover 1998). Hobcraft and Little (1984) also extended this work with their focus on fertility exposure analysis. These proximate determinants were considered as theoretically strong and highly plausible in elucidating the relationship between the level of fertility and both biological and behavioural factors, but further research using individual-level micro data demonstrates that additional factors beyond these proximate determinants also operate to explain the variations fertility outcomes and levels (for a review see: Balbo et al. 2013; Mills et al. 2011).

## The emergence of a biodemographic approach to fertility

Recognition of the importance of biological or genetic determinants underpinning demographic behaviour in this area of research began to flourish in the late 1990s. Biodemography in general grew as a fruitful interdisciplinary approach, first applied in the area of longevity and mortality studies and the relationship between fertility-longevity interactions (Vaupel et al. 1998; Wachter and Finch 1997; Wachter 2008; see also Baudisch this volume).

Udry (1994, 1996) promulgated some of the earliest calls to include a biodemographic approach to study fertility and related behaviour in contemporary demographic research. His landmark article in 1996 went beyond the data constraints of that period and the prevalent application of behaviour-genetic models (e.g., Plomin 1994) to hypothesize a series of probable biosocial relationships at the individual micro-level and societal macro-level. This included consideration of additive, indirect and interaction effects between biological (hormonal, genetic) and social factors and environments. He likewise acknowledged that the behavioural choices or motivations to have children were likely guided by biological predispositions such as genetics, hormones, neurological structure or neurotransmitters. Such predispositions could be examined by proxy. One example could be using personality as a proxy, which has been shown to operate as an antecedent to childbearing motivations and fertility outcomes (e.g., Miller 1992, 1994; Miller and Pasta 1994; Miller et al. 2010).

Another pivotal hypothesis introduced in Udry’s (1996) paper outlined how changing social arrangements could alter the proportion of variance in fertility behaviour that is biologically controlled. In other words, in times when there are high normative and social constraints on the ‘proper’ timing of first birth and number of children a woman or couple should have, the less the variance in their social behaviour should be controlled by biological forces. Conversely, in the period of the second demographic transition where individuals and couples are less socially constrained and have considerable choice, biological forces should have a stronger influence on behaviour. Adopting this argumentation, biological predispositions should be more important than in the past. This idea was adopted in further research to understand cohort differences, which we describe shortly (e.g., Kohler et al. 2002, 2006).

At the start of the 2000s, the biodemographic approach to fertility began to flourish with two interdisciplinary books appearing, *Offspring: Human Fertility Behaviour in Biodemographic Perspective*, co-edited by Wachter and Bulatao (2003) and *The Biodemography of Human Reproduction and Fertility* co-Edited by Kohler and Rodgers (2003). Both books provided broad coverage of the topic that goes beyond this limited review (e.g., endocrinology, neuroscience) and remain essential reading for this topic. Perhaps the most influential research and approach in fertility until now, however, has been the application of behavioural genetics models, which we turn to now.

## Behavioural genetics approach

In this section we first examine early studies that focused on establishing whether fertility was in the genes by producing heritability estimates in family and twin studies. We then turn to the central findings of behavioural genetics fertility research, producing a summary of results to describe the heritability of NEB and AFB. Gene and environmental interaction at the macro-level is then touched upon, concluding with multivariate models that go beyond heritability estimates and touch upon topics related to motivation and education.

### Measuring genetic influence: is fertility in the genes?

The first questions to be answered in all behavioural genetics research are whether and to what extent genes have an influence on a trait^3^ of interest (Guo 2005). In other words, it is the basic question first asked by Kohler et al. in 1999: ‘Is fertility in the genes?’ where they adopted a twin study approach to first ask and examine this question more directly and empirically in demography, the findings of which we describe shortly.

The statistical concept used to measure genetic influences on a trait within a population is called *heritability* (see Appendix 1 for a detailed explanation), and is defined as the fraction of the overall variance of a trait in a population that is caused by (additive) genetic effects^4^ (Visscher et al. 2008). It has been argued that all traits are heritable to some extent (Turkheimer 2000) and that heritability is ubiquitous in social science (Freese 2008). However, the genetic variance component provides insight into the extent to which genetic variation in a population is associated with variation in fertility. The key point is that heritability can be estimated without measuring actual genes. For this reason, family (Fisher 1930) and most commonly twin studies (Snieder et al. 2010) are conducted.

#### *Family studies*

Traditional family-studies follow simple designs such as parent-offspring correlations. Parents and children share on average 50 % of their genes. This entails two times the observed correlations in a trait. For example, the number of children ever born (NEB) estimates the heritability assuming that the shared environment of family members does not play a role in the correlation of the NEB. Correlations in fertility are very common among family members and also increased during the Twentieth century (Murphy 1999). It should also be noted that additional fertility studies in demography have examined the impact of genes albeit indirectly via the study of the intergenerational transmission of the number of children (e.g., Anderton et al. 1987; Murphy and Wang 2001), teenage pregnancy (e.g., Furstenberg et al. 1990; Kahn and Anderson 1992) and age at first birth (e.g., Barber 2000; Steenhof and Liefbroer 2008).

The pioneering work of Fisher (1930) found an intergenerational correlation of 0.20 in the NEB in data from around 2000 British aristocrats born at the end of the nineteenth century. He therefore estimated that 40 % of the variance in the NEB within this population was attributed to genetic differences. The assumption that intergenerational correlations in fertility are entirely due to genetic effects, however, does not likely hold. In fact, we know from numerous studies in sociology and demography that shared environmental factors among family members or non-genetic inheritance of traits can lead to similarities in fertility among family members (Murphy 1999; Steenhof and Liefbroer 2008). The educational level or economic status, for example, are relatively stable across generations (Jæger 2012; van Doorn et al. 2011) and at the same time important for fertility outcomes (Mills et al. 2011). Therefore, it is necessary to separate genetic from environmental effects in families and within different societal contexts.

#### *Twin studies*

The most common way to disentangle influences of latent family factors on any trait of interest is to use the information available in twin data. Twin models follow a relatively simple and straightforward logic and until now, represent one of the best resources for evaluating the importance of genetic variation in observed traits (Boomsma et al. 2002).

Twin models facilitate the comparisons between two kinds of twins, identical or monozygotic (MZ) and fraternal dizygotic (DZ) twins, in order to quantify genetic and non-genetic environmental influences. In a classic twin design, MZ as well as DZ twins are siblings of virtually identical ages and born and raised in the same families. The siblings are consequently assumed to share common environmental influences such as their parents, the neighbourhood they grew up in and other related aspects. More importantly, MZ twins are genetically identical (i.e., share all their genotypes). DZ twins in contrast, are akin to full siblings and thus share on average only around 50 % of additive genetic effects. Similar to parent–offspring correlations, the correlation among DZ twins therefore reflects the importance of both environmental and genetic effects in families. The degree, however, to which MZ twins have a higher correlation in the trait of interest than DZ twins, reflects the fact that they are genetically more similar. The comparison of twin correlations thus already makes it possible to quantify genetic and shared environmental effects related to a particular trait (for details regarding the quantitative models to estimate these effects see Appendix 2) (Boomsma et al. 2002; Snieder et al. 2010).

There has been some criticism of the potentially problematic assumptions and practical limitations of twin studies (for critical discussions on the equal environment assumption for example, see Horwitz et al. 2003). Both twin and family designs are likewise limited for other reasons in fertility research such as the fact that they require a pair of siblings, thus excluding only children and thus individuals from low fertility families. Since dizygotic twinning also has a genetic basis, it is likely that these twins carry genes that might be important for high fertility. There might be a non-random genetic stratification which could bias variance estimates. Finally, these designs require multiple family-members which are more difficult and costly to gather than data on unrelated individuals.

### Central findings of behavioural genetics fertility research

Adopting the aforementioned behavioural genetics approach, a series of studies have focussed on predicting the heritability of number of children ever born (NEB), age at first birth (AFB), which we summarize here.

#### *Heritability of NEB*

**Fig. 1:**
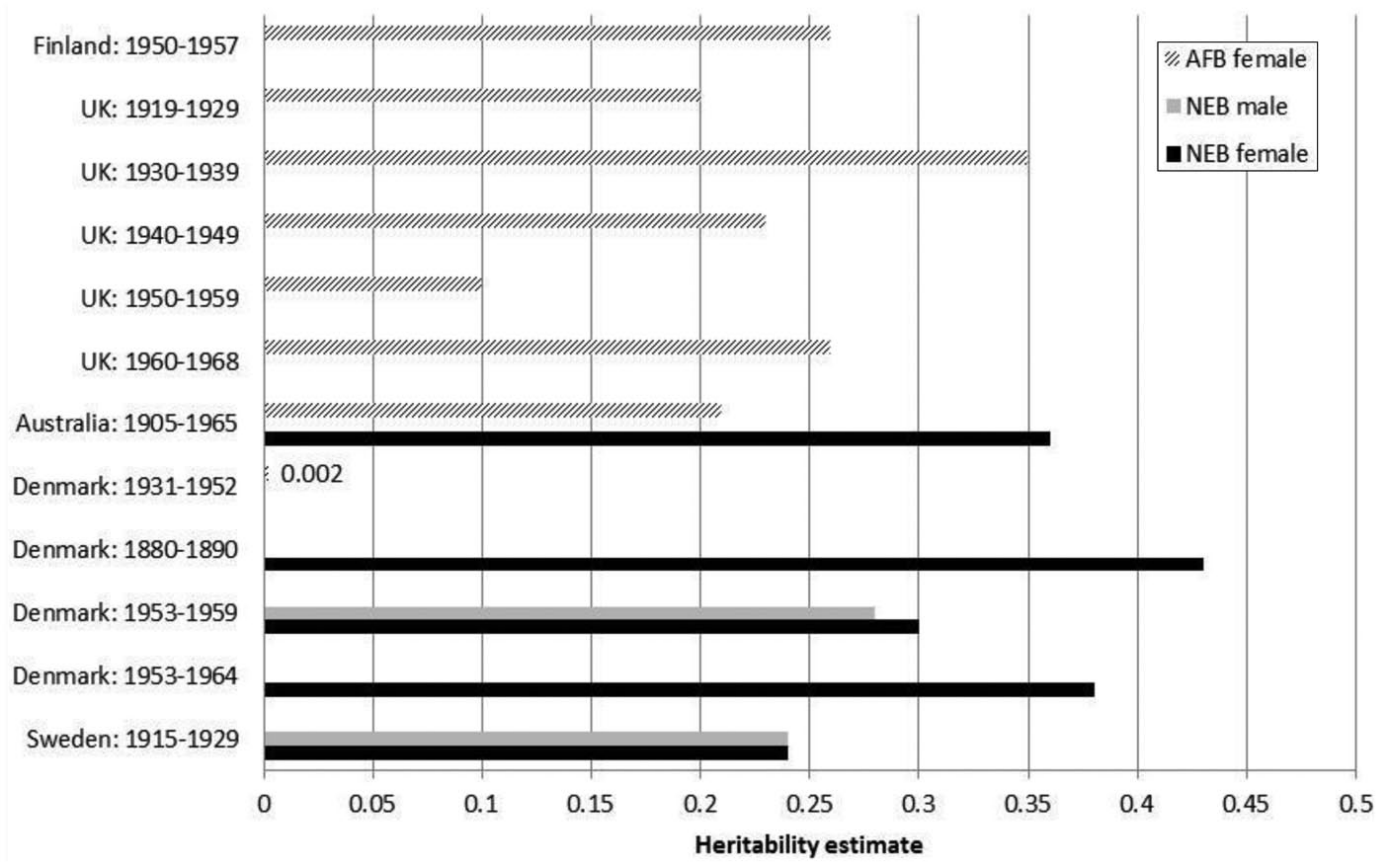
Summary of fertility heritability estimates by birth cohort and country. *AFB* age at first birth, *NEB* number of children ever born. Sources: Finland, Nisén et al. (2013); UK, Tropf et al. 2015a; Australia, Kirk et al. (2001); Denmark, Kohler et al. (1999).

Several (twin) studies provide evidence for a genetic component underlying both the tempo (AFB) and quantum (NEB) of human fertility up to a level of over 40 % (Byars et al. 2010; Kirk et al. 2001; Kohler et al. 2006; Tropf et al. 2015a). Figure 1 provides an overview of selected studies that have produced heritability estimates by birth cohort across different countries. The figure shows the estimates for women for age at first birth (AFB) and for both men and women of number of children ever born (NEB). Here we can see that heritability estimates for AFB for women range between 0.002 (Denmark 1931–1952) and 0.35 (UK 1930–1939) or in other words that up to just over 0 or 35 % of the observed variance in AFB within these birth cohorts is due to additive genetic effects, respectively (see Appendix 1). For NEB for women, the range is 0.24 (Sweden 1915–1929) and as high as 0.43 (Denmark: 1880–1890) and for men, the range for the same trait is between 0.24 (Sweden 1915–1929) and 0.28 (Denmark 1953–1959). The figure shows that there is considerable variation in heritability estimates across time, between countries and the sexes, a point we return to later.

Focusing on NEB, a study by Rodgers et al. (2001) investigated genetic and environmental influence on the NEB among female and male twins from Denmark born between 1953 and 1959. They found that twin correlations among MZ pairs (~ 0.30) are more than twice as high as those of DZ twin pairs (0.13) for both males and females. The structural equation models (see Appendix 2) estimate heritability for both sexes of around 0.30, meaning that 30 % of the observed variance in the NEB within these birth cohorts is due to additive genetic effects. Both non-additive genetic and shared environmental effects were not significant, and therefore the remaining 70 % of the variance can be attributed to non-shared environmental effects between the siblings, respectively measurement error. These findings are almost perfectly replicated in a recent study of Swedish twins born between 1915 and 1929 (Zietsch et al. 2014).

Not only in fertility research, but behavioural genetic research in general shows that considerable variation comes from non-shared environmental factors. Unique environmental factors appear to be the main source of variation; amongst others, the partner of the individuals play an important role (Kohler and Rodgers 2003). Additionally, such results may suggest gene × environment (G × E) interaction in reference to fertility, since, at least in twin models, this would lead to an inflation of non-shared environmental effects/measurement error. G × E refers to the situation where genetic effects that are associated with a different trait are dependent upon the variability in the environment or when genes modify an organism’s sensitivity to particular environmental features (e.g., Shanahan and Hofer 2005). A particular context can trigger or compensate for a genetic vulnerability and improve adaptation. The findings until now suggest that biodemographic research on fertility needs to continue to embrace demographic and sociological research that has uncovered vital predictors of the non-shared environment.

#### *Heritability of AFB*

The age at first birth (AFB) has been assessed in a recent study by Nisén et al. (2013) on Finnish twins born between 1950 and 1957. For men they found that common environmental factors of siblings—and not their genes—are the central underlying factors to explain resemblance in the AFB among brothers. For women, in contrast, they estimate heritability in the AFB at 0.26, which is also in line with studies from the UK (Tropf et al. 2015a) and Australia (Kirk et al. 2001) (see Fig. 1). However, the AFB turns out to be a challenge to study, since other studies in the U.S. (Neiss et al. 2002) and Denmark (Rodgers et al. 2008) show no significant effect on the AFB of women.

A core issue in the analysis of AFB is the handling of right-censored observations (i.e., individuals that did not have a first birth by the last time of observation). Opposed to the commonplace practice of including right-censored cases in demography and sociology (e.g., Mills 2011), it is uncommon to deal with censored information in genetic studies and childless women are generally excluded from the sample (Byars et al. 2010; Neiss et al. 2002; Nisén et al. 2013; Rodgers et al. 2008). Using data from the TwinsUK, Tropf et al. (2015a) empirically examined whether the inclusion of censored information in a Tobit model affects heritability estimates compared to the classic models. Results show strong and non-systematic differences between both kinds of models suggesting that the comparison of these research designs has to be critically reconsidered. Readers might also wonder how it is possible that heritability estimates for the same trait might differ between birth cohorts and countries, a topic to which we turn to now.

### Gene and environment (G × E) interaction at the macro-(population) level

Different heritability estimates for a trait between different populations are not necessarily pointing purely to methodological issues, but can be informative in substantive terms. As described previously, researchers such as Udry (1996) hypothesized that there is likely an interaction with biological and societal level factors at the macro- or population level. In fact, one of the most common misconceptions about genetic studies is that the heritability of a trait within one population can predict the heritability in the same trait in another population—even though similarities in estimates can be remarkable for physical traits such as height (Visscher et al. 2008). First, heritability is a ratio of genetically caused variance over the overall variance. Therefore, changes in the overall variance in a trait can change heritability independent of the genetic variance component, or in other words—heritability is a population parameter (see Appendix 1). Second, the genetic variance component depends on the genetic endowment, for example, of allele^5^ frequencies within a population. Third, and presumably most important for fertility, genes always only encode predispositions for a trait, but environmental conditions determine whether these dispositions become manifest (Guo 2005). Thus, genes and the environment can interact. Replication in different temporal and spatial settings is thus pivotal to gain insight about this interplay.

Gene × environment (G × E) interaction has also been examined in the study of NEB. Using data on the historical Danish twin cohorts including virtually every twin pair born since 1870, Kohler et al. (1999) and Kohler et al. (2002) report large changes in heritability estimates for the NEB for cohorts born during the past centuries. Particularly, after the strong fertility decline of the First Demographic Transition at the end of the nineteenth-century and the Second Demographic Transition in the second half of the twentieth-century, heritability reached a moderate level of 0.40—while getting close to zero in the interim phases.

As described in more detail previously, this fits with the explanation hypothesized by Udry (1996) who described changes in the influence of genes on reproductive behaviour in terms of gene × environment interaction with factors such as societal norms constraining choice and behaviour. Udry argued that genetic predispositions for fertility gain importance in environments that are less restrictive in their fertility choice. At the same time, genetic predispositions play a minor role in restrictive social contexts such as strong normative rules about the timing and number of children, war or economic crisis.

Following Udry’s (1996) hypotheses, Kohler et al. (1999) associated the observed peaks in heritability with the environmental changes during the Demographic Transitions. In particular, there were improvements in economic, medical and hygienic conditions during the First Demographic Transition, with the Second Demographic Transition characterized by the introduction of the pill and cultural transformations relaxing fertility norms triggering genetic expressions (Van de Kaa 1987). A recent study corroborates this reasoning, applying an extension of the family model on a large scale database containing 100,000 sibling pairs from the Dutch Province of Zeeland born between 1810 and 1870 (Bras et al. 2013). These findings not only show a rise in heritability during the first Demographic Transition, but they present evidence that this rise was especially true for women in more urban areas or with a liberal religious climate, thus underscoring the idea that individual freedom triggers genetic influences on the NEB.

Attention to gene and environmental interactions have also been found in studies that focus on the AFB. A study by Tropf et al. (2015a) analysed changes in the genetic influence on the AFB across birth cohorts from 1920 to 1964 of female UK twins. They found large swings in heritability co-varying with strong environmental upheavals. While heritability is low or insignificant for birth cohorts who started childbearing during the end of World War II or the economic crisis of the 1970s and 1980s, it rises to around 0.40 for individuals who started childbearing in times of changing mores and the sexual freedom in the 1950s and 1960s.

Although the focus until now in this review has been on NEB and AFB, other related fertility traits have also been examined. Genetic influences have been explored for the age at first dating or marriage (Mealey and Segal 1993), age at first sexual intercourse (Dunne et al. 1997), number of sexual partners (Guo et al. 2008) and likelihood of unprotected sexual intercourse (Daw and Guo 2011). One of the strongest genetic effects was found in a twin study examining the first attempt to have a child among the Danish twins, measured in retrospective interviews of the first attempt to conceive. In this study, up to 50 % of the variance in that trait was explained by genetic differences (Kohler et al. 1999; Rodgers et al. 2001). This raises the question of how different fertility traits relate to each other and how far the same genes or the same environment is important for different fertility traits.

### Beyond heritability estimates: multivariate models of fertility behaviour

Several studies engage in more complex (twin) modelling (e.g., Kohler et al. 2011) estimating, for example, bivariate genetic models. Following the same logic as in classic twin studies, it is possible to estimate the extent to which the same genetic and environmental factors are important for two different traits. If trait 1 of twin 1 predicts trait 2 of twin 2, the correlation of traits runs in families and therefore there is a common etiology of the traits within the family. If this cross-trait-cross-twin correlation is higher for MZ twins than for DZ twins, the common etiology is partly genetic. If the genetic or environmental correlation between two traits is 1, all genetic variance in trait 1 and 2 has a common base. If the genetic correlation is 0 the genetically based variance between trait 1 and 2 are independent. For a more detailed empirical explanation, refer to Kohler et al. (2011).

#### *Decision-making and fertility motivations*

One area of research has focused on decision-making and motivations. Rodgers et al. (2001) combined information on the NEB in the Danish twin cohorts with the first attempt to become pregnant. They found firstly, that the age at first attempt to conceive is heritable for both men (0.35) and women (0.52) and secondly, that around 10 % of the genetic variance in the NEB is related to the genetic variance in the first attempt to conceive. This study can be seen as key evidence that part of the genetic dispositions influencing our realized fertility is not due to physical traits, but mediated by conscious decision making.

A more recent study by Miller et al. (2010) empirically demonstrated that a portion of the genetically influenced fertility behaviour is related to the motivational precursors to fertility. Examining both timing and NEB and using the NLSY79 in the U.S., they conducted both uni- and multivariate behavioural genetic analyses. The central finding was that there are genetic additive effects that operate through desires to have a certain number of children that affect both the timing of the next child that is born and the ultimate NEB. They also link these affects to gender role attitudes but find no genetic variance associated with either child-timing intentions or educational intentions. This was an extension of previous work such as that by Miller (1994) and Pasta and Miller (2000), showing that both positive and negative childbearing motivations were significantly heritable and that the fertility outcomes we observe are antecedent to desires in the motivational process. This builds on Miller’s (1994) Traits-Desires-Intentions-Behaviour (TDIB) theoretical framework.

#### *Education and fertility*

Education—and particularly women’s education—is a central predictor of particularly the timing of first and subsequent births (Mills et al. 2011). Multivariate genetic models have been applied to explore the relationship between education and fertility with respect to its common genetic and environmental base.

The aforementioned study on Finnish twins (Nisén et al. 2013) shows that the negative association of education with the chance of having any children among women is largely genetically based. The same accounts for the positive association between education and the chance to have any children for men, suggesting that different genes are important for men and women concerning fertility. Using more complex models, two studies from the U.S. (Neiss et al. 2002) and Denmark (Rodgers et al. 2008) test whether education mediates the negative relationship between intelligence and AFB in a behavioural genetic framework. Results show that education partially mediates the effect of intelligence on AFB in standard models, but that this mediation is not significant once a model is fitted that takes latent family factors (environmental and genetic ones) into account. In other words, differences in intelligence, education and the AFB emerge from variance between families, not differences between siblings within the same family. Such findings may cast doubt on the assumption that education causally affects the timing of childbearing and thus has the potential to challenge the widely accepted assumption that educational expansion of women is the main explanation for the massive postponement in the AFB during the second half of the Twentieth Century (Balbo et al. 2013; Mills et al. 2011; Ní Bhrolcháin and Beaujouan 2012).

## Molecular genetics approach

Virtually all of the fertility-related research conducted within biodemography has adopted a behavioural genetics approach until now. Recent advances in molecular genetics, however, offer new possibilities. Simply put, the main distinction is as follows. Whereas behavioural genetics focused on whether fertility is in the genes and to what extent it was heritable, molecular genetics attempts to isolate where the genetic variants are located, in addition to a focus on the structure and function of DNA. We now briefly describe current and on-going research in this area, which are extended further in the final section on future promising research areas.

### Fertility-related candidate gene studies

An initial approach in this area of research adopted what is now commonly referred to as the candidate gene approach. This is an approach that has an a priori hypothesis about the underlying biological pathway of a trait and immediately focuses directly on a gene or set of genetic markers^6^. The research design is straightforward and is easily conducted, generally only on one population with relatively small samples. Researchers equipped with both the relevant social science indicators and measured genetic variants could compare a sample of the ‘treatment’ or affected group that had the genetic marker(s) compared to the ‘control’ or unaffected group. This approach with pre-determined hypotheses was familiar to the type of hypothesis driven research often conducted in the social sciences.

Although there are no direct candidate-gene studies on the fertility outcomes related to timing (e.g., AFB) or number of children (NEB) to date, several fertility-related studies have been conducted on sexual behaviour and contraceptive use. These studies examined the impact of certain pre-hypothesized genetic polymorphisms^7^, generally in relation to hypotheses related to risky behaviour and sensation seeking and linking it to the dopamine receptor or serotonin transporter. The fertility-related research to date in demography and sociology has been conducted on one sample, which is the U.S. National Longitudinal Study of Adolescent Health (AddHealth).

Guo and Tong (2006) conduct both a behavioural genetic twin analysis and a molecular genetic approach to examine the association between the age at first intercourse and the 48-bp repeat polymorphism in the dopamine receptor D4 gene (DRD4). They found evidence from the twin approach that there was a genetic basis of timing of first intercourse and that those with an any-3R genotype had a higher risk of intercourse than those from the control group (other/other or any-4R genotype). This also held after controlling for covariates (e.g., ethnicity, socioeconomic status, gender, family structure etc.). They concluded that genes played a role in the timing of first sexual intercourse.

Halpern et al. (2007) examined 717 heterozygous young adult sibling pairs to focus on sexual behaviour and the number of sex partners. They studied three genetic polymorphisms including DRD4 (in the dopamine D4 receptor), DRD2 (dopamine D2 receptor) and 5HTT (serotonin transporter promoter), which was hypothesized to be linked to sensation seeking. They found that DRD4 was not related to sensation seeking and number of sex partners and that the A1 DRD2 and 5HTT allele (see note 9) went against their expectations, was small in magnitude and was associated with fewer sexual partners. The researchers acknowledged the limitations of the approach and difficulty in examining the complex outcomes of sexual relationships.

Guo et al. (2008) examined white males and engaged in a gene-environment interaction analysis with a focus on the number of sexual partners and concluded that the 9R/9R genotype relative to the Any10R genotype in the dopamine transporter gene (DAT1) had a protective effect against having a higher number of sexual partners. The effect, however, differed by cognitive ability and the school environment, leading the authors to conclude that there was a more complex interplay at hand between the outcome, social context, individual influences and genetic predispositions.

Another study by Daw and Guo (2011) drawing from the same data examined the genetic associations related to adolescents’ contraceptive use, again linking it to the notion of higher rates of impulsivity. Adopting a similar approach, they concluded that the genetic variants in the dopamine transporter gene DAT1*9R/10R, dopamine receptor gene DRD2 (*A1/A2 and A2/A2) and the monoamine oxidase gene MAOA*R were associated with a lack of contraceptive use (i.e., unprotected sexual intercourse).

Although these results have been path-breaking, the candidate-gene approach has more recently been highly criticized (for a review see for example Ioannidis 2005; Duncan and Keller 2011). The main problem is that there has been high selectivity not only in the samples, but also the results that have been published to date. As Duncan and Keller (2011) demonstrate in the field of psychiatry, which adopted this approach earlier and more rigorously, these candidate-gene studies rarely replicated on new samples. A problem that has now been acknowledged in other disciplines is that there was a publication bias, often with only positive associations accepted (or sent for review) for publication. Another core issue was the often small sample size which led to extremely low statistical power (Ioannidis 2005). For this reason, these types of studies are viewed with scepticism until results can be replicated on additional and larger samples.

#### *Genome Wide Association Studies (GWAS)*

After the backlash of candidate-gene studies, Genome Wide Association Studies (GWAS) emerged as a promising new approach to connect genetic variants to the outcomes of interest (Zhao and Chen 2013). GWAS refers to hypothesis-free testing without any a priori assumptions about either the biological pathway or a particular location (Nolte et al. 2010). It likewise embraces the fact that there are multiple genes (polygenic) and pathways associated with fertility that is difficult to specify in advance with our current state of knowledge. The approach capitalizes on recent developments in microarray technology to identify the associations between specific traits and genetic variants across the entire genome. It rapidly scans markers across the complete genome of many people to find genetic variations associated with a particular trait. GWAS are possible due to the completion of the Human Genome Project in 2003 and the International HapMap Project in 2005 which provided the basic tools to find the genetic contributions of traits. As with other genetic data available until now, it is necessary to have the DNA from each participant in the study, often via a blood or saliva sample. Each person’s DNA is then placed on tiny microarray chips and scanned on automated laboratory machines. These machines quickly overview each person’s genome for strategically selected markers of genetic variation, referred to as SNPs (single nucleotide polymorphisms).^8^ A GWAS therefore runs millions of separate regressions on the phenotype (outcome) of interest across the entire genome. Due to the large number of SNPs that are tested in GWAS, an association must achieve a stringent threshold of statistical significance (P < 5 × 10^−8^) in order to be considered as validated. A positive association refers to the case where there is a greater frequency of a genetic variant in individuals with that trait compared to those in the control group (i.e., absence of trait). The association identifies a genomic region and not a specific causative mutation that might be involved in the development of the trait or behaviour.

The computational GWAS approach remains promising for social science research due to the fact that it overcomes some of the mistakes inherent in candidate-gene studies in the past and due to the fact that the often evasive biological pathway of the trait does not need to be specified in advance. It is also the only technique currently available that has the potential to discover novel genes which could then be used in further more reliable follow-up studies and provides directions of where researchers need to search and potential biological pathways to pursue. It also allows population stratification to be controlled, which remains a key issue in avoiding bias and misinterpretation of results in this type of research (see Wray et al. 2013 for a review).

A promising first application in the social sciences examined the genetic variants to predict educational attainment on more than 120,000 genotyped individuals by Rietveld et al. (2013a) as part of the Social Science Genetics Association Consortium (www.ssgac.com). At the time of writing this review, Mills and her research team at the University of Oxford are currently leading a large consortium to engage in the first ever genome-wide association search (GWAS) and meta-analysis of reproductive choice (age at first birth; number of children), conducted in both men and women in over 50 data sets, with the results replicated in additional datasets in a large sample.

Although GWAS are able to narrow in on where to look in the genome, recent research has criticized (Hirschhorn 2009) that the validated SNP associations explain only a small portion of the genetic variance or heritability of the phenotype that has been estimated from classic family and twin studies, often referred to as the ‘missing heritability’ problem (Maher 2008). Despite the fact we are currently working towards isolating SNPs conferring the genetic variation of reproductive behaviour in the previously mentioned GWAS, we anticipate that this might not be the entire story of the genetic architecture of reproductive outcomes. The expectation is that for a complex outcome such as reproductive choice, we will be able to explain some—but not all—of the genetic heritability with a GWAS. Furthermore, as discussed momentarily in the conclusion, interaction with genes and the environment are likewise crucial. Due to this general problem, also found in relation to more complex disease outcomes and traits, additional promising methods have been developed.

Although no GWAS has been published as of yet of the reproductive choice variables we often study in demography and sociology, several studies have shown promising results for fertility-related outcomes related to infertility and the reproductive life span (menarche, menopause). Previous research has successfully demonstrated that there is a genetic component to reproduction with over 70 genome-wide association studies (GWAS) published for 32 traits and diseases associated with reproduction found (see Montgomery et al. 2014). This includes identification of genes such as those related to age at menarche (Sulem et al. 2009; Lui et al. 2009; Elks et al. 2010), menopause (Snieder et al. 1998; Stolk et al. 2009, 2012; Perry et al. 2013; He et al. 2009), and endometriosis (Painter et al. 2011).

The results from a recent study among a natural fertility population of Hutterites by Kosova et al. (2012) examining male fertility traits and isolating 41 SNPs, likewise shows promising areas to examine further. In this study, nine of the SNPs were associated with reduced sperm quality, providing a further link to potential biological pathways in men. Due to a strict religious doctrine in the Hutterite community, the variation in non-genetic factors is minimized between individuals, allowing them to confirm the presence of a significant genetic component in the natural variation of fertility. The use of these types of results, particularly in interaction with the socio-environment, could lead to a new understanding of fertility as we know it within the social sciences.

### Evolutionary anthropology and biology

The behavioural genetics modelling of human fertility has obviously not only been exclusively an interest for social scientists, but widely prevalent among evolutionary biologists and anthropologists. The question of evolution was initially more of a question of surviving until one reproduces, since only those who survived were able to transmit their genes to the next generation. However, due to improvements in hygiene and the subsequent reduction in prenatal, infant and child mortality, whether and how many children an individual bears has become are important question to understand evolution. If genetic variants are associated with fertility success, this means that some genetic variants will be more frequent in subsequent generations than others and therefore indicate natural selection and evolution (Stearns et al. 2010).

This line of reasoning, however, theoretically predicts that fertility as well as other behaviour important for the transmission of genes to the next generation show low or no significant genetic variation (Kimura 1958) within a population, because evolution should have already trimmed the differences over time. As mentioned at the start of this review, it has been assumed that this so-called Fundamental Theorem of Natural Selection (FTNS, Fisher 1930) is the reason why genetically informed fertility research has been neglected for some decades (Kohler et al. 1999; Rodgers et al. 2001).

However, empirically this FTNS turns out to be only partly true. On the one hand, we do observe a much higher heritability for morphometric traits (for example height: ~ 0.80 for human) than for traits such as fertility (Visscher et al. 2008). On the other hand, fertility traits do still show significant heritability. Kirk et al. (2001) for example analysed the heritability of the NEB in reference to the NEB of the peers in a cohorts, to determine in how far genes influence the relative reproductive advantage in a population among Australian female twins. They find a heritability of 0.36, indicating that modern societies are still under natural selection.

#### *Evidence for natural selection*

The genetic correlation of a trait with NEB furthermore indicates whether a specific trait is under natural selection. Findings from the Framingham Heart Study, for example, suggest that the same genes influencing the NEB are negatively correlated with a number of traits, among others height, cholesterol levels, systolic blood and the AFB (Byars et al. 2010). Consequently, the authors expect selective changes in the disposition for these traits for subsequent generations (e.g., that the female US population under study will shrink in body-size).

The negative association between NEB and the AFB partly explains that we observe a correlation between both traits but also leads to a prediction of a decrease in the AFB across generations (Byars et al. 2010; Kirk et al. 2001). Such findings are in line with studies on natural populations such as from Milot et al. (2011). They observed in a historical population of natural fertility (high fertility norms in a stable environment) in Canada that the AFB decreased around 3 years within the past 140 years as a response to natural selection. Kirk et al. (2001) argue that in contemporary times, compared to the past, more psychological and social traits are important for the NEB and the AFB and therefore assumed to be under natural selection.

### Conclusions and discussion: limitations and fertile future research frontiers

The aim of this review was to examine the current state of knowledge in the area of the biodemography of fertility. After touching upon the different terminology and underlying reasons for the lack of research into this field we turn to the early foundations of approaches that included both behavioural and biological determinants of fertility. The current review is the first to summarize findings from behavioural genetic research on this topic which show that there is a clearly genetic component to fertility outcomes, with both AFB and NEB having a heritability ranging from zero to levels of just over 40 %.

Since heritability is a population parameter, heritability of a trait within one population cannot predict the heritability within another (see Visscher et al. 2008). This leads us to acknowledge the importance of social science research of the environment and promising studies that have examined swings in heritability with environmental changes and choice in fertility decisions. Other fruitful lines are studies that have gone beyond heritability estimates to probe into more complex multivariate models of fertility behaviour such as first attempts to conceive a child (Rodgers et al. 2001) and motivational precursors to fertility (Miller 1994; Pasta and Miller 2000; Miller et al. 2010).

Research using a molecular genetic approach first adopted a candidate-gene approach, which have been criticized and requires further replication, but provides an initial window into possible mechanisms and approaches that might be interesting to pursue in future research. More promising approaches are hypothesis-free methods to find genetic variants related to AFB and NEB via a Genome Wide Association Study (GWAS). Since it is highly implausible that even the best social scientist could specify the biological pathway and isolate particular genetic variants in advance, this approach offers an alternative. We then reviewed results of several studies in evolutionary anthropology and biology that showed that contemporary populations might still be under natural selection (e.g., Kirk et al. 2001; Byars et al. 2010; Milot et al. 2011).

### Discussion and areas of future research

#### *Towards truly interdisciplinary work and quality control*

A central challenge to adopting a biodemographic approach in fertility research has been the lack of training and understanding of very different concepts in the different disciplines. Entering a different field has imminent danger, with the social sciences already making some fundamental mistakes via naïve candidate gene studies that have produced false positive findings that will likely fail to replicate. This concern was emphasized by Wachter (2008, p. 1503), who argued this key challenge, which is: “the need to keep a high standard of quality control, as interdisciplinary researchers step out beyond their original base of expertise.” This means not only borrowing the techniques or data from a particular field, but actively engaging with researchers in those fields and following training to ensure high quality standards. But this is an approach that begs for precious time and resources often unavailable for researchers and requires them (their employers and funders) to possess the ability to understand delayed gratification and long-term thinking, since investments into a new discipline may take years to bear fruit. In the current sphere of competitive research and focus on production, many researchers—and particularly young scientists—may be unable or reticent to make these kinds of long-term investments. Interdisciplinary training programmes and centres could be a valuable solution to train the next generation of truly interdisciplinary scholars.

### Fertile frontiers of new substantive research topics

#### *Towards more complex models of gene × environment (G × E) interaction*

It is highly likely that there are multiple ways in which the socio-environment can moderate the genetic endowment of individuals. There are two main ways in which genetic dispositions may influence human fertility. First, there can be a direct effect on physiological characteristics (e.g., fecundity, age at menarche, age at menopause). Second, biological predispositions may affect the processes of decision-making and life course planning, consciously and subconsciously (Kohler et al. 2006). Genes provide the potential for a trait, but environmental conditions determine whether that potential will be realized. The most promising future research will therefore be that which attempts to go beyond the examination of only direct effects of genetic and socio-environmental factors to exploring the combined interaction, which is likely greater than their independent effects. It would be interesting to explore how the socio-environment moderates gene expression. It is imaginable that triggers such as stress might activate certain hormones and other bodily functions more prevalent in individuals with a particular genetic endowment which in turn could inhibit their fecundability. We also know that social compensation, in the form of social capital and strong networks can result in individuals being able to realize their initial fertility intentions (Balbo and Mills 2011).

#### *Epigenetics*

The growing interest and study of epigenetics is another relevant consideration for future research (Landecker and Panofsky 2013). This relates to the dynamic nature of genes and how chemical bases of gene expression are influenced by DNA methylation. It is plausible that socio-environmental exposures might actually alter gene expression, such as the study by Cameron et al. (2005) which demonstrated that the maternal behaviour of rats (grooming, nursing) affected gene expression among offspring in the brain regions that control defensive and reproductive behaviours. Another recent study on rats also showed epigenetic silencing or the inhibition of DNA methylation as a mechanism underlying the neuroendocrine control of female puberty (Lomniczi et al. 2013). In humans, factors such as breast-feeding and parental interaction could be potentially interesting factors to study.

#### *Sex differences*

Almost identical results in heritability estimates for men and women e.g. (Rodgers et al. 2001) might suggest that the same genes are important for male and female fertility. However, the study of Nisén et al. (2013) shows that genes predicting childlessness in women are associated with low education among women and high education among men. Therefore the genetic architecture of fertility might differ considerably between the sexes.

#### *A population paradox*

Evolutionary predictions that there is a tendency to have children at a younger age over time seem to contradict widespread observations of fertility postponement at the population level in many European countries. Models from biologists suggest that the AFB decreases across generations (Byars et al. 2010; Kirk et al. 2001), while we observed a massive postponement during the second half of the past century (Mills et al. 2011). Potential explanations for this population paradox are, amongst others, gene environment interaction or additive environmental effects such as the educational expansion or the introduction of the pill overriding smaller effects of natural selection. However, it becomes obvious that an integrative approach between biological and social science is necessary to answer questions of human fertility and evolution.

#### *Integrating new data and methods from quantitative genetics and reproductive medicine*

As mentioned at the onset of this review, a central reason for the lack of biodemographic research in the area of fertility has been a lack of data that combines good social science indicators with data that has biomarker and genetic data in large samples. The recent significant reduction in the costs of collecting, storing and processing this type of data will hopefully result in new sources for the future. Although considerable research has been done, many questions remain open. The fact that heritability is a population parameter and therefore can vary over time and space requires replication of heritability estimates in different societies of interest.

To break new frontiers in fertility research using genetic information, methodological advances such as the ACE-β has been developed that bridge behavioural genetic and econometric approaches. This approach combines insights from econometrics and behavioural genetics by integrating the MZ-fixed effects approach as a direct link between phenotypes into the structural equation model. It therefore potentially identifies a causal link between two traits additional to genetic and environmental endowment—particularly if a valid instrumental variable can be found (these and related approaches are detailed in Kohler et al. 2011). More importantly, in the realm of molecular genetics, actual genetic data is being collected as well as further development of statistical tools (Yang et al. 2010, 2011). Using actual genetic information, it becomes possible to determine the heritability of one as well as the genetic correlation between two traits without family data—which is often problematic to gather and requires strong assumptions in the modelling (Conley et al. 2013). This likewise raises the level of flexibility in testing gene environment interactions and sex differences.

Additional techniques have also been developed within quantitative genetics to try to explain more of the variance predicted in behavioural genetics models or in other words to address the ‘missing heritability’ problem. Several methods combine many SNPs to aggregate the collective effect and achieve a higher level of predictive power such as polygenic risk scores (Purcell et al. 2009; Mandemakers et al. 2014) or genome-wide complex trait analysis (GCTA) (Yang et al. 2010). In a recent GCTA application, Tropf et al. 2015b demonstrated that 10 % of the variance in NEB and 15 % of the variance in AFB can be directly related to measured SNPs from the whole genome.  Finally, other promising applications include using genes as instrumental variables to go beyond establishing association to making causal inferences. Endogeneity problems are often difficult to disentangle, with these approaches offering at least a partial solution (e.g., Barban et al. 2014; Lawlor et al. 2008).

A central conclusion from this review is that biological and genetic factors are relevant in explaining and predicting fertility traits. The socio-environment, which demographers and social scientists have studied until now, however, still appears to account for the major part of the observed variance. Studying the interplay between genes and the environment, new data sources and integration of new methods will be central to more effectively understanding and predicting fertility trends. Findings, such as the common genetic base of the first attempt to have a child and NEB furthermore suggest that genes not only mediate physical but also psychological traits and conscious decision making. In the coming years we anticipate that the field and interest will grow as we become able to isolate which genetic variants are important for fertility, understand their biological functions and how they interact with the socio-environment.

## Appendix:

**Appendix 1**: Heritability—a population parameter

The concept to capture the genetic component underlying a trait is named heritability and is the proportion of the genetically caused variance (*V*_*G*_) over the overall variance of the trait (*V*_*P*_ phenotype) in a specific population (for details see Visscher 2008):

$${{H}^{2}}=\frac{{{V}_{G}}}{{{V}_{P}}}$$

Whereas the phenotypic variance is the sum of genetic and environmental variance.

$${{V}_{P}}={{V}_{G}}+{{V}_{E}}$$

The genetic component can be furthermore differentiated into additive (*V*_*A*_) and non-additive (*V*_*NA*_, epistatic and dominant) effects.

$${{V}_{G}}={{V}_{A}}+{{V}_{NA}}$$

Generally, for most quantitative traits—a trait that shows continuous variation (Turkheimer 2000)—it is assumed that non-linear effects play only a minor role. Specifically in the fertility literature, evidence for epistatic or dominant effects is negligible. Therefore, the genetic component underlying a trait is commonly quantified in terms of narrow sense heritability and when we use heritability in this article we mean:

$${{h}^{2}}=\frac{{{V}_{A}}}{{{V}_{P}}}$$

In other words, heritability quantifies the genetic variance component. Narrow heritability can be understood as the *R* ^2^ from a regression of all genetic alleles of the genome on a trait, whereas the effects of the alleles are constraint to be additive (Rietveld et al. 2013b).

**Appendix 2**: Estimating variance components in twin models

A naïve approach to quantify the proportion of explained variance by additive genetic effects is to compute the correlations of a trait separately for MZ and DZ twin pairs. Since they are assumed to share family environment to the same extent and MZ are twice as similar as DZ twins with regard to their genes, narrow sense heritability is two times the difference of the intra-group correlations of MZ and DZ.

$${{h}^{2}}=2\times \left( \text{r}\left( \text{MZ} \right)\text{-r}\left( \text{DZ} \right) \right)$$

The effect of the common environment of the twins (*c* ^2^) is therefore the pairwise correlation of MZ minus the heritability.

$${{c}^{2}}=\text{r}\left( \text{MZ} \right)-{{h}^{2}}$$

Variance that is unexplained by these factors is due to non-shared environmental effects from outside or even within the family (Pike and Kretschmer 2009; including measurement errors: for details (see Snieder et al. 2010).

Based on this logic, structural equation modelling (SEM) has become standard in twin research. Above correlations have low power and large standard errors and do not make use of information available in variances and covariances. SEM furthermore provides goodness of fit statistics enabling the testing of alternative models and identifying assumptions—for example whether there are dominant genetic effects (Snieder et al. 2010). Extensions of the twin model to other relatives as well as other family designs are possible.

## Footnotes

1 We are aware that this is a simplification of the term fitness and that more nuanced measures differentiated between absolute fitness (ratio between number of individuals with genotype after selection to those before selection) and relative fitness (average number of surviving progeny of a particular genotype compared to average of surviving progeny of competing genotypes after a single generation). Fitness is defined as a propensity or probability of the expected average number of offspring in a particular group or class of individuals and not only the actual number of offspring produced.

2 Bongaarts (1982) adapted the definition of postpartum infecundability to represent the combined effect of postpartum amenorrhea and abstinence.

3 This research often refers to a trait or phenotype, which in the social sciences is generally referred to as an outcome or dependent variable.

4 An additive genetic effect is the estimate of quantitative inheritance such that the combined effects of genetic alleles (see note 5) at two or more gene loci (see note 6) are equal to the sum of their individual effects. In other words, it is the quantitative change in a trait that is associated with substituting one allele (i.e., one genotype) with that of another allele within one population. Humans (and other organisms) have two alleles for each trait and an allele is an alternative form of a gene (i.e., one member of a pair) that is located at a specific position on a specific chromosome. These different codings determine the distinct traits that are passed on from parents to offspring.

5 An allele is an alternative form of a gene (i.e., one member of a pair) that is located at a specific position on a specific chromosome. The DNA codings determine the distinct traits that are passed from parents to offspring.

6 A genetic marker is a uniquely identifiable, short DNA-sequence.

7 A genetic polymorphism describes the existence of different forms of DNA sequences at a position (locus) in a population (see also note 9).

8 DNA consists of around 3 billion pairs of nucleotide molecules and each can be indexed by its location in the gene. There is no variation for most of these nucleotides across individuals; rather, we differ on our polymorphisms and hence the focus on SNPs, which occur once every 100–300 nucleotides. A single gene can contain hundreds of SNPs.
